# Assessment of predictive validity and feasibility of Edmonton Frail Scale in identifying postoperative complications among elderly patients: a prospective observational study

**DOI:** 10.1038/s41598-020-71140-5

**Published:** 2020-09-07

**Authors:** Yingke He, Lydia Weiling Li, Ying Hao, Eileen Yilin Sim, Kai Lee Ng, Rui Lee, Mattheaus ShengJie Lim, Ruban Poopalalingam, Hairil Rizal Abdullah

**Affiliations:** 1 Division of Anesthesiology and Perioperative Medicine, Singapore General Hospital, Outram Road, Singapore, 169608 Singapore; 2 Department of Anaesthesia and Intensive Care, Changi General Hospital, 2 Simei Street 3, Singapore, 529889 Singapore; 3 Health Service Research Centre, Singapore Health Services, Outram Road, Singapore, 169856 Singapore; 4 Division of Nursing, Singapore General Hospital, Outram Road, Singapore, 169608 Singapore; 5 Yong Loo Lin School of Medicine, National University of Singapore, 10 Medical Drive, Singapore, 117597 Singapore; 6 Duke-NUS Graduate Medical School, 8 College Road, Singapore, 169857 Singapore

**Keywords:** Risk factors, Geriatrics, Rehabilitation

## Abstract

Frailty is defined as diminished physiological reserve predisposing one to adverse outcomes when exposed to stressors. Currently, there is no standardized Frail assessment tool used perioperatively. Edmonton Frail Scale (EFS), which is validated for use by non-geriatricians and in selected surgical populations, is a candidate for this role. However, little evaluation of its use has been carried out in the Asian populations so far. This is a prospective observational study done among patients aged 70 years and above attended Preoperative Assessment Clinic (PAC) in Singapore General Hospital prior to major abdominal surgery from December 2017 to September 2018. The Comprehensive Complication Index (CCI) and Postoperative Morbidity Survey (POMS) were used to assess their postoperative morbidity respectively. Patient’s acceptability of EFS was measured using the QQ-10 questionnaire and the inter-rater reliability of EFS was assessed by Kappa statistics and Bland Altman plot. The primary aim of this study is to assess if frailty measured by EFS is predictive of postoperative complications in elderly patients undergoing elective major abdominal surgery. We also aim to assess the feasibility of implementing EFS as a standard tool in the outpatient preoperative assessment clinic setting. EFS score was found to be a significant predictor of postoperative morbidity. (OR 1.35, *p* < 0.001) Each point increase in EFS score was associated with a 3 point increase in CCI score. (Coefficient b 2.944, *p* < 0.001) EFS score more than 4 has a fair predictability of both early and 30-day postoperative complications. Feasibility study demonstrated an overall acceptance of the EFS among our patients with good inter-rater agreement.

## Introduction

Frailty is defined as the biologic syndrome of a decreased physiological reserve, resulting from the cumulative declines of multiple organ systems, which predisposes one to adverse outcomes when exposed to stressors such as surgery^1,2^. As frailty is the composite effect of multiple organ deficiencies, it has been shown to be better in predicting hospitalization stay, complications and morbidity among the elderly patients when compared to individual assessment of medical comorbidity or functional status^1,3^.

There is an increasing number of non-cardiac surgeries done among elderly people worldwide^4^. The prevalence of frailty is estimated to be at 10–15% among those between 65 to 75 years old. This frequency increases to more than 40% among those beyond 85 years old^3,5–7^. However, despite several frailty assessment tools being available, routine assessment is not commonly done prior to elective surgery^8–10^. Furthermore, a patient's preoperative optimization strategy rarely incorporates the formal diagnosis of frailty^8,11,12^. This is an important observation as frailty is associated with poorer outcomes among surgical patients, such as the increased risk of infection, postoperative delirium, postoperative mortality, prolonged hospital stay and healthcare-related cost^4,13–16^. While multiple implementation barriers may contribute to this, the choice of the assessment tool is an important consideration^8^. In the perioperative setting, a frailty assessment tool should be highly predictive of postoperative outcomes, and able to highlight areas amenable for optimization before surgery. Moreover, it should be both feasible and simple to administer by the non-geriatricians in the busy preoperative assessment setting.

The current frailty assessment tools can be divided into the phenotype model such as frailty phenotype which focuses on functional assessment or the cumulative deficit model such as frailty index giving deficits in the domains of comorbidities^14^. However, both tests are time-consuming when incorporated into routine pre-operative assessment and they provide clinicians little information regarding areas for modification^2,17^. The Edmonton Frail Scale (EFS), being a performance-based multidimensional frailty assessment tool, is designed to aid in the assessment and screening of frail elderly patients in the primary care setting, as well as at bedside, by non-geriatricians in the western population^18^.

EFS consists of 11 questions over nine different domains such as cognition, social support, mood, nutrition, and functional performance. The EFS was found to be a valid measure of frailty as the result was well correlated with the clinical impression of geriatricians through detailed history and physical examination^18,19^. EFS has also been shown to be able to predict operative risk when used as a screening tool in the Caucasian population^11^. However, little evaluation of its use has been carried out in the Asian population so far, which has one of the fastest aging populations in the world.

The primary aim of this study is to assess if frailty measured by EFS is predictive of postoperative complications among elderly patients who are undergoing elective major abdominal surgery in an Asian healthcare system. Furthermore, we aim to assess the feasibility of implementing EFS as a standard tool in the outpatient preoperative assessment clinic setting.

## Methods

### Study design

This is a single-center, prospective observational study conducted in Singapore General Hospital (SGH), which is the largest tertiary public hospital in Singapore with over 1,700 beds. This study consisted of two separate parts, namely assessment of predictive validity of EFS for postoperative complications and assessment of the feasibility of EFS in the preoperative outpatient setting. Informed consent was obtained from all patients and the study protocol was approved by the Institutional Review Board (SingHealth CIRB 2018/2548) prior to the start of the study. The study was performed in accordance with relevant guidelines and regulations.

Patients aged 70 years and above, who attended SGH Preoperative Assessment Clinic (PAC) prior to elective major abdominal surgery between December 2017 and September 2018 were enrolled in this study. Major abdominal surgery is defined as an intra-peritoneal surgery, expected to last more than 2 h, or with an anticipated blood loss greater than 500 mL including surgeries in the disciplines of general surgery (upper gastrointestinal and hepatobiliary surgery), colorectal surgery, urological and gynecological surgery.

#### Assessment of predictive validity of EFS

Five trained nurses in the PAC who are blinded to other details related to the patient’s planned surgery (type of surgery, patient’s medical comorbidities, surgical plan etc.) administered the questionnaire to patients who met the recruitment criteria in English, Chinese, Malay or Tamil in the same standard manner. The total EFS score was calculated by the nurses at the end of the assessment. Patient’s demographic data, clinical laboratory data, comorbidities as well as 30-day postoperative complications were searched from the institution’s clinical information system (Sunrise Clinical Manager, Allscripts, Illinois, USA) by two assessors (HYK and LWL).

#### Assessment of the feasibility of EFS in the preoperative setting

We define the feasibility of EFS based on patients’ acceptability, duration of time taken to complete the questionnaire and inter-rater variability. Patients’ acceptability of EFS was evaluated using the QQ-10 questionnaire. The time taken for those patients to complete EFS was also recorded.

### Measurement

#### Assessment of frailty

##### Edmonton Frail Scale (EFS)

The EFS is an 11-question questionnaire which analyses nine domains of frailty (cognition, general health status, functional independence, social support, medication use, nutrition, mood, continence, functional performance), with the maximum score being 17, representing the highest level of frailty. The patients were then classified into five categories based on their EFS scores as suggested on the official EFS website (Edmontonfrailscale.org), ranging from Fit (0–3), Vulnerable (4–5), Mildly frail (6–7), Moderately Frail (8–9) and Severely frail (≥ 10).

#### Assessment of predictive validity of EFS

Postoperative outcome data were entered into the electronic medical system by the primary surgical team. Postoperative morbidity data were analyzed using the Comprehensive Complication Index (CCI)^20^ and Postoperative Morbidity Survey (POMS)^21^ to assess the predictive validity of EFS in the preoperative setting.

##### Comprehensive Complication Index (CCI)

The Comprehensive Complication Index (CCI) is a measure currently widely adopted to assess postoperative complications^20^. Based on the widely established Clavien–Dindo classification (CDC), it integrates into a single formula all complications of varying severity, ranging from 0 (uneventful) to 100 (death). Compared to CDC, which includes only the most severe complications, it captures all postoperative morbidity by severity over time, producing a continuous scoring variable^22^. 1-month follow-up was selected to allow better capture of all postoperative complications.

##### Postoperative Morbidity Survey (POMS)

Postoperative Morbidity Survey (POMS) is a method used to describe short-term morbidity after major surgery. It is a nine domain tool based on organ systems. POMS was assessed on postoperative days 3, 5, 7. For each domain, the presence or absence of morbidity is recorded based on objective criteria. A score of 0 will be given if no complication is present and 1 for any complication present under each domain on that particular postoperative day^21^.

#### Assessment of the feasibility of EFS in the perioperative setting

##### QQ-10 questionnaire

QQ-10 is a 10-item questionnaire, developed to assess the face validity, feasibility and utility of other healthcare questionnaires^23,24^. 6 out of 10 questions assess how patients value the healthcare questionnaire (helped me communicate about my condition, relevant to my condition, easy to complete, included all the aspects of my condition I am concerned about, was enjoyable, would be happy to complete as part of routine care), while 4 out of 10 assess the burden of the questionnaire to the patient (too long, embarrassing, complicated, upset me). The patient scores each question from a scale of 0 to 4, with 0 being “strongly disagree” and 4 being “strongly agree”. The mean value score is calculated as an average score of the 6 questions assessing the value of the questionnaire from all patients, while the mean burden score is calculated from the four burden questions.

##### Time taken to complete EFS

Time taken to complete the EFS (in seconds) in PAC was recorded for patients recruited in the feasibility study by five clinic nurses.

##### Inter-rater variability of EFS

Inter-rater reliability was assessed using weighted Kappa and Bland Altman plot^25^.

### Sample size calculation

#### Assessment of predictive validity of EFS

The mean difference of CCI across the groups of frailty (non-frail, vulnerable and frail) was about 11–12 in our pilot study. Based on the effect size obtained, an estimated sample size of at least 14 patients in each group will allow us to significantly detect the mean difference of CCI across the groups of frailty with Type 1 error of 0.05 and Type II error of 0.2 using ANOVA analysis, assuming a dropout rate of around 20%.

#### Assessment of the feasibility of EFS in the perioperative setting

Based on our prior clinical experience, we estimated the mean value score obtained from QQ-10 questionnaire to be around 80%. 30 patients’ data will allow us to have an interval estimate (confidence level 95%) of mean value score with precision of 15%.

### Statistical analyses

Data collected were analyzed using R 3.5.1 (R Core Team (2018). R: A language and environment for statistical computing. R Foundation for Statistical Computing, Vienna, Austria. URL https://www.R-project.org/). Descriptive data are expressed as mean with standard deviations for continuous variables and frequencies (percentages) for categorical variables.

One-way ANOVA was performed to assess the differences in continuous variables including age, BMI, length of stay as well as CCI and POMS scores among the five categories of frailty groups. Chi-square /Fisher’s exact test was performed to investigate the correlation between level of frailty and categorical variables, e.g. gender, race, surgical disciplines, types of surgery.

A multivariable linear regression model was applied to assess the correlation of EFS score with CCI adjusted by patients’ demography and surgical characteristics. A multivariable generalized linear mix model was performed to estimate adjusted odds ratio of EFS score in predicting the presence of 30-day postoperative complications (measured by total POMS score more than 0). The area under the receiver operating characteristic (ROC) curve which is plotted by the true positive rate (sensitivity) against the false positive rate (1-specificity) was used to evaluate performance of using EFS to predict the presence of early and 30-day complication and quantify the cut-off EFS score using Youden’s index. Weighted Kappa and Bland Altman plot for 2 raters were used to assess inter-rater reliability.

A *p* value of < 0.05 was used to determine statistical significance.

### Ethical approval and consent to participate

The study protocol and written informed consent were approved by the Institutional Review Board (SingHealth Centralized Institutional Review Board 2018/2548, Singapore health service group).

### Consent for publication

Written informed consent was obtained from all patients.

## Results

### Demographics

**Figure 1:**
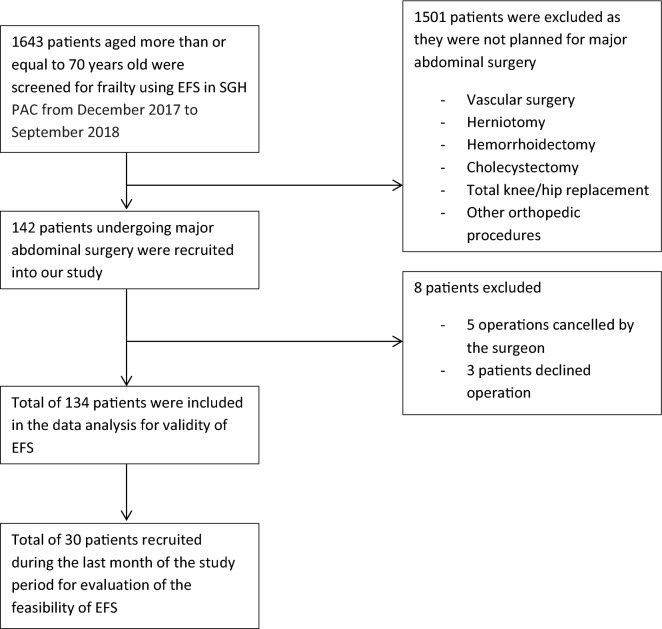
Study flowchart.

A total of 142 patients who met our inclusion criteria were recruited into the validity study. Eight of them were excluded from the data analysis as the operation was either cancelled by the surgeon or by the patients themselves (Fig. 1). The remaining 134 patients undergoing major abdominal surgery were included in the analysis of the validity study and 30 patients out of this cohort into the feasibility study, as none of them refused to participate. Based on their EFS scores, 62 of them are fit (EFS ≤ 3) (46.3%), 33 of them are vulnerable (EFS 4–5) (10.4%). 14 patients (10.4%) are mildly frail (EFS 6–7), 12 are moderately frail (9%) and the remaining 13 are severely frail (9.7%) (Table 1). Patients in the frail group are older but there is no significant difference in terms of BMI, gender, race as well as surgical discipline they belonged to among the different groups of patients. In addition, a higher proportion of frail patients underwent open surgery compared to the non-frail group who had more laparoscopic procedures done for similar surgical conditions (*p* = 0.013) (Table 1).

**Table 1:** Comparison of demographics among patients with different degrees of frailty.

	Fit	Vulnerable	Mildly frail	Moderately frail	Severely frail	*p* value
Number of patients	62 (46.3)	33 (24.6)	14 (10.4)	12 (9)	13 (9.7)	
**Age mean (SD)**	74.2 (4.2)	76.8 (4.9)	77.5 (6.2)	77.7 (5.7)	78.3 (6.9)	0.029
**Gender (freq (percent))**						0.077
Male	39 (58.2)	14 (20.9)	6 (9)	4 (6)	4 (6)	
Female	23 (34.3)	19 (28.4)	8 (11.9)	8 (11.9)	9 (13.4)	
**Race (freq (percent))**						0.454
Chinese	57 (46.3)	31 (25.2)	12 (9.8)	11 (8.9)	12 (9.8)	
Malay	3 (60)	1 (20)	0 (0)	0 (0)	1 (20)	
Indian	0 (0)	1 (50)	1 (50)	0 (0)	0 (0)	
Others	2 (50)	0 (0)	1 (25)	1 (25)	0 (0)	
**BMI (kg/m**^**2**^**)** **(mean(sd))**	23.2 (5.3)	24.3 (4.7)	23.7 (3.7)	24.7 (4.2)	25.3 (4.2)	0.545
**Surgical discipline (freq (percent))**						0.066
Urology/gynaecology	30 (63.8)	10 (21.3)	4 (8.5)	1 (2.1)	2 (4.3)	
Colorectal	16 (41)	12 (30.8)	4 (10.3)	4 (10.3)	3 (7.7)	
General surgery	16 (33.3)	11 (22.9)	6 (12.5)	7 (14.6)	8 (16.7)	
**Type of surgery (freq (percent))**						0.013
Open	15 (29.4)	13 (25.5)	8 (15.7)	7 (13.7)	8 (15.7)	
Laparoscopic	47 (56.6)	20 (24.1)	6 (7.2)	5 (6)	5 (6)	

### Validity of EFS to predict postoperative risk

Both CCI scores and POMS were used to study short-term postoperative morbidity. A statistically significant difference in mean CCI score was found between fit, vulnerable, mildly frail, moderately frail and severely frail group. (*p* < 0.001) (Table 2). A similar result was demonstrated when POMS analysis was performed at postoperative day 3, 5 and 7. (*p* < 0.05) A step-wise increase in mean CCI and POMS score was also observed across the five groups of patients. Furthermore, frail patients have a longer length of hospital stay compared to fit or vulnerable elderly patients going through similar operations (*p* = 0.034) (Table 2).

**Table 2:** Comparison of postoperative complications among patients with different degrees of frailty.

	Fit	Vulnerable	Mildly frail	Moderately frail	Severely frail	*p* value
Number of patients	62 (46.3)	33 (24.6)	14 (10.4)	12 (9)	13 (9.7)	
CCI (mean(SD))	11.1 (12)	16.1 (12.1)	24.5 (16.4)	28.6 (17.1)	42.1 (27.4)	< 0.001
POMS day 3 (mean(SD))	1.3 (1.5)	1.9 (1.7)	1.9 (1.3)	2.3 (1.5)	3.2 (2)	0.012
POMS day 5 (mean(SD))	0.5 (0.9)	0.8 (1.1)	1.2 (1.4)	1.4 (1.3)	3.1 (2.3)	0.003
POMS day 7 (mean(SD))	0.2 (0.4)	0.4 (0.7)	0.8 (1.4)	0.9 (1.3)	2.5 (2.4)	0.005
LOS (days) (mean(SD))	7.4 (6.5)	10 (11.4)	15.1 (12.8)	12.7 (8.4)	17.9 (17.8)	0.034

Regression models were then constructed to assess the performance of EFS in predicting postoperative complications as measured by both CCI and POMS. Total POMS score more than 0 was used as a surrogate marker for the presence of any postoperative complications within 30 days. EFS score was found to be a significant predictor of postoperative morbidity in both models after adjusting for other confounders including age, gender, race, surgical disciplines and surgical technique (open or laparoscopic surgery). (OR 1.35, p < 0.001) (Table 3). Each point increase in EFS score was associated with a 3 point increase in CCI score (Coefficient b 2.944, *p* < 0.001) (Table 4).

**Table 3:** Variables associated with presence of 30-day postoperative complications (measured by TOTAL POMS score > 0 within 30 postoperative days) using generalized linear mix model (age was excluded due to small number range resulting in convergence issue in model fitting).

	Odds.ratio (95% CI)	*p* value
**EFS.SCORE**	1.35 (1.18, 1.57)	< 0.001
**Gender**		
Male	Ref	
Female	0.42 (0.2, 0.83)	0.015
**Race**		
Chinese	Ref	
Non-Chinese	4.21 (1.2, 17.6)	0.031
**BMI**	0.95 (0.88, 1.01)	0.121
**Surgical discipline**		
General surgery	Ref	
Urology/gynaecology	0.31 (0.13, 0.7)	0.006
Colorectal	0.45 (0.19, 1.03)	0.061
**Type of surgery**		
Lap	Ref	
Open	1.63 (0.77, 3.57)	0.18

**Table 4:** Variables associated with presence of 30-day postoperative complications (measured by CCI score) using linear regression model.

	Coefficient b (95% CI)	*p* value
**EFS.SCORE**	2.944 (2.012, 3.877)	< 0.001
**Age**	− 0.047 (− 0.541, 0.448)	0.852
**Gender**		
Male	Ref	
Female	− 9.53 (− 14.471, − 4.588)	< 0.001
**Race**		
Chinese	Ref	
Non-Chinese	0.053 (− 8.864, 8.971)	0.991
**BMI**	− 0.179 (− 0.686, 0.328)	0.485
**Surgical discipline**		
General surgery	Ref	
Urology/gynaecology	− 12.268 (− 18.268, − 6.267)	< 0.001
Colorectal	− 8.229 (− 14.231, − 2.228)	0.008
**Type of surgery**		
Lap	Ref	
Open	3.98 (− 1.463, 9.422)	0.15

**Figure 2:**
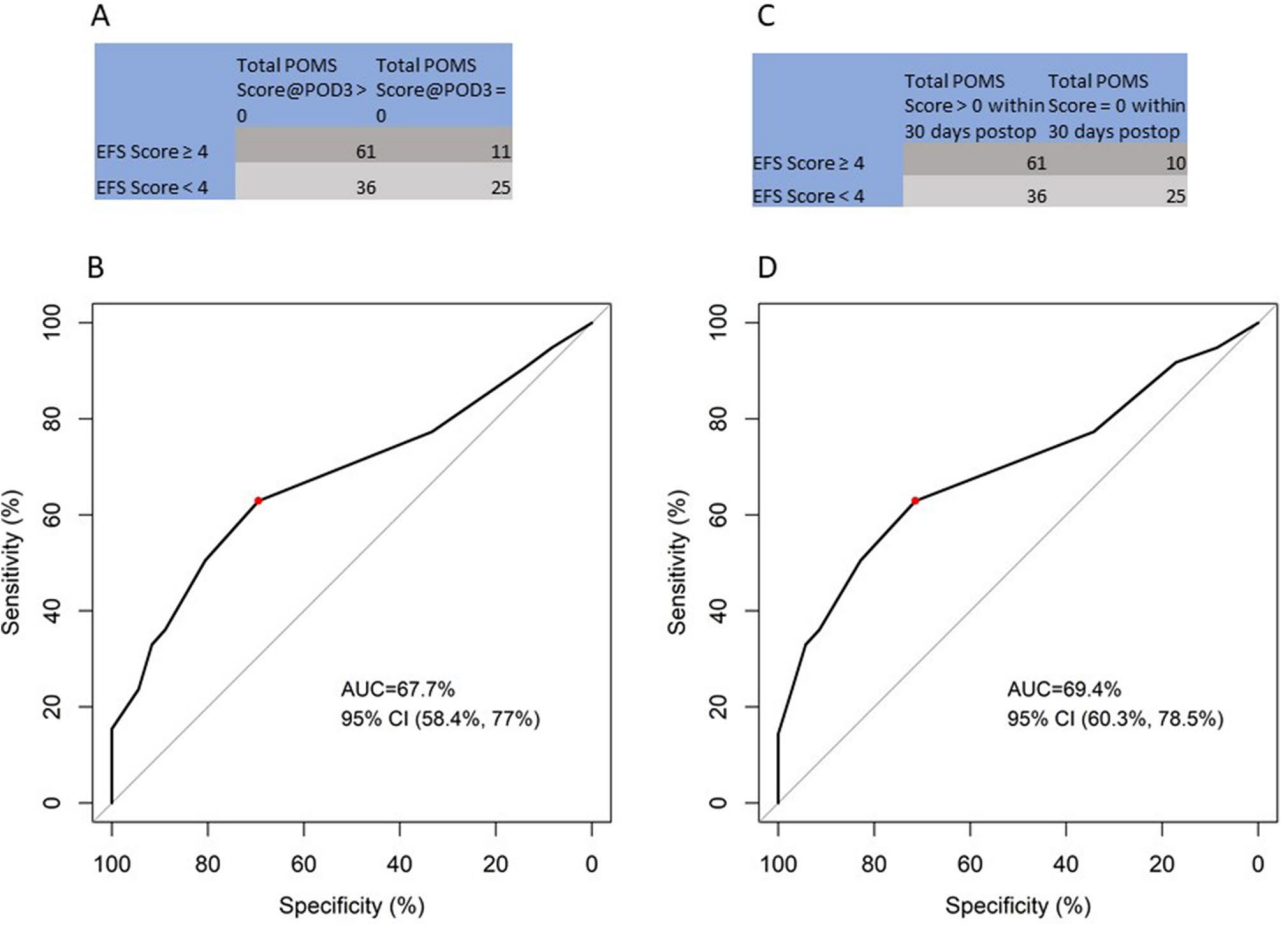
Cutoff of EFS score for predicting presence of postoperative complications (**A**) Table showing choice of EFS cut off value to predict early postoperative complications (measured by POMS > 0 @ postoperative day 3) (**B**) ROC curve of choice of EFS cut off value to predict early postoperative complications (measured by POMS > 0 @ postoperative day3) (**C**) Table showing choice of EFS cut off value to predict 30-day postoperative complications (measured by Total POMS Score > 0 in within 30 postoperative days) (**D**) ROC curve of choice of EFS cut off value to predict 30-day postoperative complications (measured by Total POMS Score > 0 in within 30 postoperative days).

ROC curve was plotted to find an optimal cut-off value that can help to predict both early (measured by POMS score > 0 at postoperative day 3) and 30-day (measured by total POMS score > 0) postoperative complication. In both ROC curves, frailty defined as EFS score more than 4 was found to have a fair predictability demonstrated by the area under the curve (AUC) of 67.7% and 69.4% respectively (Fig. 2).

Feasibility analysis of EFS in the outpatient preoperative clinic setting.

**Figure 3:**
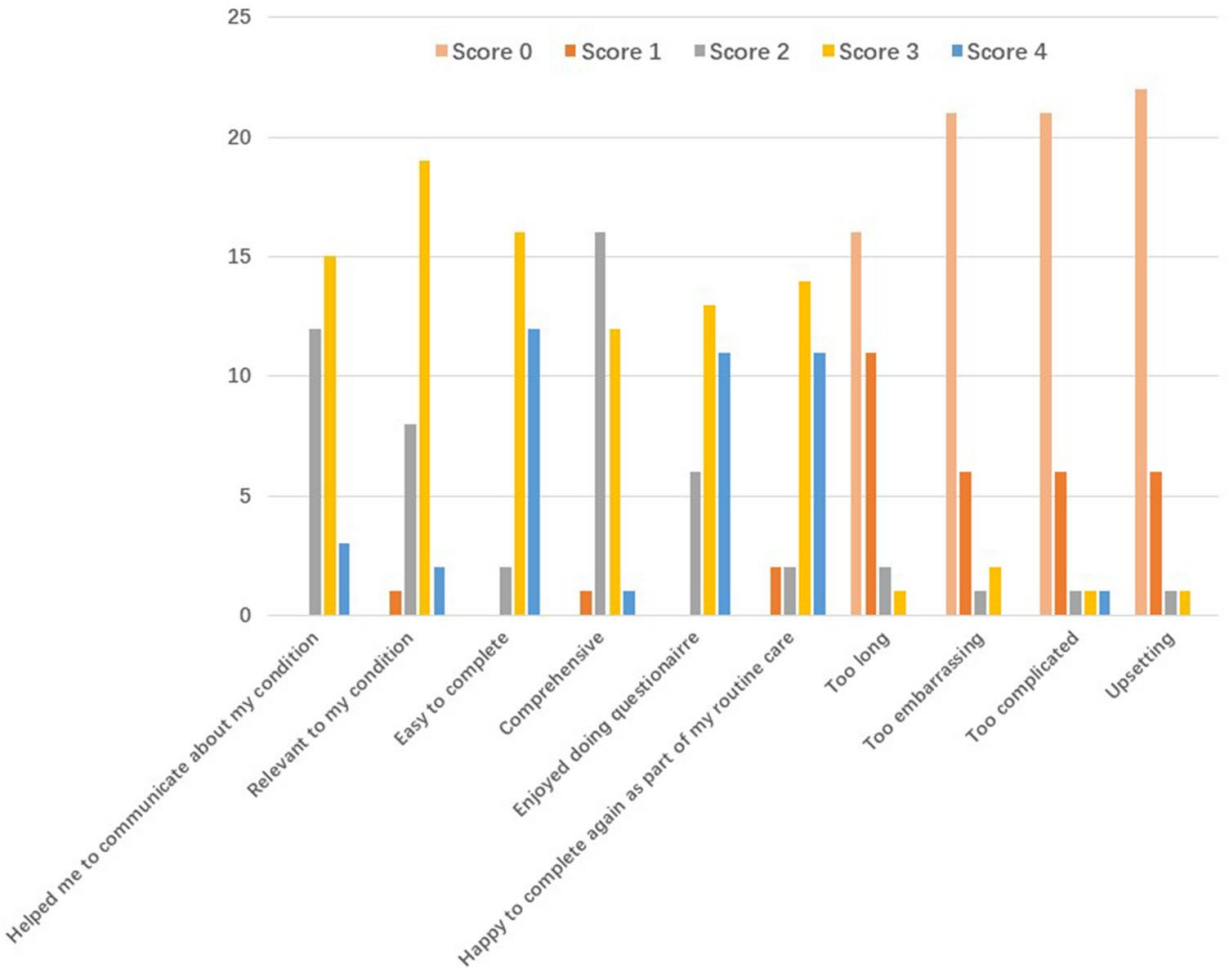
QQ-10 questionnaire result. X axis shows the content of each question (value questions in blue colour and burden questions in red colour) and Y axis shows the number of patients giving a particular score for that question. (0-strongly disagree, 1-mostly disagree, 2-neither agree nor disagree, 3-mostly agree, 4-strongly agree).

30 patients with a mean age of 76 years old took part in the QQ-10 questionnaire to understand their acceptability of EFS. The overall mean value score was 72% (SD 11.9) with a mean burden score of 12% (SD 17.1). The exact distribution of patients’ answers to each question is shown in Fig. 3. The high value score and low burden score show an overall acceptance of the EFS among our patients.

**Figure 4:**
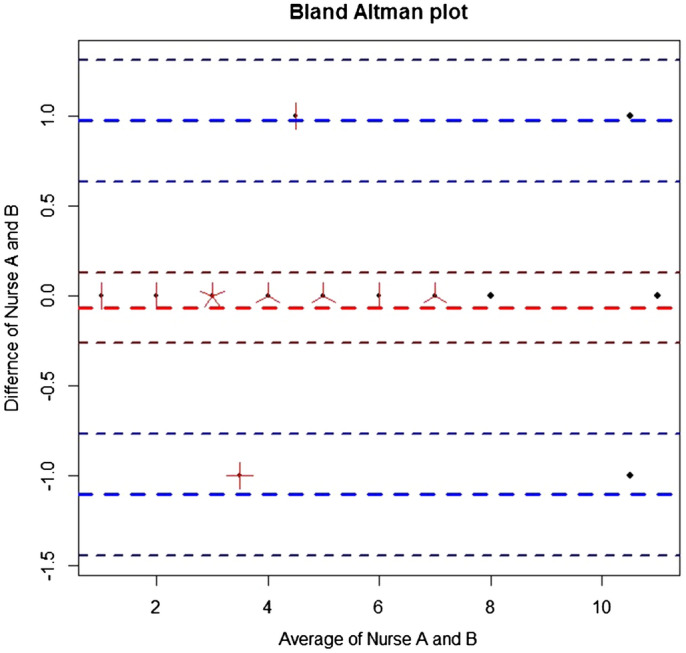
Bland Altman plot showing inter-rater agreement of EFS.

The average time taken for those 30 patients to complete EFS was 225 s (SD 57). Weighted Kappa for 2 raters was 0.973, which demonstrated almost perfect inter-observer reliability. Bland Altman plot between 2 nurses assessing the same patient also demonstrated good agreement with a mean difference of 0.07 (95% CI − 1.1, 0.97) (Fig. 4).

## Discussion

Our data, from a single-center study, support that EFS is able to predict operative risk in elderly patients undergoing major abdominal surgery. We demonstrated that frail patients measured by higher EFS scores have greater postoperative complications risk as measured by both higher POMS and CCI scores (OR 1.35). Secondly, we propose to define frailty in Asian surgical population using EFS cut-off score of 4 and above to predict postoperative morbidity. Our ROC curve using cut-off score of 4 shows a fair performance in predicting the presence of both early and 30-days postoperative complications. Furthermore, we have demonstrated that the use of EFS in the outpatient preoperative assessment clinic is feasible and well accepted by elderly patients. It can be completed quickly and has minimal inter-rater variability. These features suggest that it is feasible and appropriate for EFS to be incorporated into the routine workflow of a busy preoperative assessment clinic.

Frailty is recognized as one of the strongest preoperative predictors of postoperative complications in one of meta-analysis study recently^26^. Having a clear understanding of postoperative recovery trajectories is essential to help clinicians conduct appropriate discussions about treatment plans with patients and family^27^. The value of Comprehensive Geriatric Assessment (CGA), the current gold standard for frailty assessment among geriatricians, in predicting frailty in colorectal cancer patients was shown by a study conducted by Krishnan et al., but its applicability in the busy preoperative clinic setting is questionable^28^. In another retrospective observational study, Saxton et al. demonstrated the predictive role of frailty measured by Frailty Index in identifying high risk patients planning for abdominal surgery^29^. However, as the items in Frailty Index were obtained through either history or physical examination, it provides little information on the specific deficit domain which the clinicians need to work on. The absence of a universally accepted measurement tool prevents frailty assessment from being incorporated into routine preoperative practice.

An assessment tool for use in the clinical setting should consider the two main purposes of preoperative identification of frailty: risk stratification, and identification of factors for potential modification^10^. In the acute care hospital population, multi-dimensional frailty assessment tools like EFS provide more information on the severity and the types of deficits experienced by a patient^10^. Previous studies have shown that EFS captures appropriately every area of frailty, with a high degree of correlation between EFS scores and CGA as well as other frailty scales^18,30^. Our results in the preoperative setting of patients planning for major abdominal surgery are consistent with findings by Dasgupta et al. that increasing EFS scores were associated with increased length of hospital stay and postoperative complications independent of age in orthopedic patients^11^. Similar results have also been reported in urological, cardiac as well as colorectal surgery patients in North America and Europe^30–32^.

Previous work on the perioperative use of EFS has been focused on its predictability of postoperative mortality and length of hospital stay, but little attention has been given to the exact EFS score that could help with risk stratification in the surgical population^11,33,34^. Currently, there is no consensus on the use of EFS cut-off scores to define non-frail, vulnerable and frail among the clinicians globally. The most widely used classifications include a two cut-off point system dividing the elderly patients into non-frail (EFS < 5), vulnerable (EFS 6–7) and frail (EFS > 8)^31,35^ and a four cut-off point system which separate the population into five categories like our study^11,36^. By having a more delicate classification system, we felt that the five-category system allows clinicians to risk stratify the patients with higher specificity based on their degree of frailty. This will facilitate clinical decision making and proper risk counselling when encountering frail patients in the preoperative clinic. In our study, the estimated prevalence of frailty in elderly surgical population is around 29% (EFS score more than 6). Study conducted by Hoover et al. estimated the prevalence of frailty in the Canadian community to be 22% based on community health survey^37^. The difference in prevalence can be possibly due to the larger number of frail elderly in the surgical population compared to the general public. Our ROC analysis demonstrated for the first time that frailty defined as EFS score of 4 and above in the surgical population could give perioperative clinicians fair predictability of increased postoperative complication risk. This is consistent with the result described by Dasgupta et al., who has demonstrated that EFS score more than 4 is associated with a significantly higher positive likelihood ratio when predicting postoperative complications^11^.

A good assessment tool in a clinical setting should be acceptable to the elderly surgical patients, easy to use by non-geriatricians and not burdensome to the healthcare system. Compared to other multidimensional tools such as CGA and Comprehensive Assessment of Frailty (CAF) score which are either too complex or too lengthy restricted for research use, EFS can be completed within 5 min and easily followed by non-geriatricians. The study by McIsaac et al. have suggested that almost all elderly patients are willing to participate in a frailty assessment before going for major surgery^34^. The high value score and low burden score obtained from the QQ-10 data suggested that our patients had a pleasant experience with EFS questionnaire during their preoperative visit and found it as making a positive impact on their health care. This makes EFS an ideal tool to be incorporated into routine preoperative frailty assessment.

The strength of the study includes the prospective study design with a complete follow up. The 1-month follow up period includes some of the subacute post-operative complications, allowing us to better validate the use of EFS in the preoperative setting. The use of two different postoperative morbidity measurements (CCI and POMS) with different grading systems helped to reduce the potential assessor bias making the conclusions more objective. The use of a separate patient’s acceptability measurement tool (QQ-10) further enhanced our knowledge about the clinical usefulness of EFS.

This study has several limitations. The single-center study design and the relatively small number of sample size may restrict its generalizability. Secondly, the outcome assessors were not blinded to the EFS score of the recruited patients. However, since the postoperative outcome data were entered into the electronic medical system by the surgical team who were unaware of the EFS score, this is unlikely to result in any observer bias. Further studies should be conducted to validate the ability of the EFS cut-off score proposed in predicting postoperative complications in larger patient populations, as well investigating the frailty domains that are particularly associated with poor outcomes to facilitate preoperative rehabilitation programs.

## Conclusion

Our study has demonstrated that frailty measured by EFS is able to predict postoperative complications in elderly patients undergoing elective major abdominal surgery. EFS scores are closely correlated with postoperative morbidity as measured by POMS and CCI scores. Frailty defined as EFS score of 4 and above gives clinicians a fair predictability of an increase in post-operative risk. We have also shown that EFS is a feasible tool to be incorporated as a standard assessment in the outpatient preoperative clinic setting, with high patients’ acceptance and low inter-rater variability.

## Data Availability

The dataset analysed during the current study are available from the corresponding author on reasonable request.

## Abbreviations

EFS: Edmonton Frail Scale
CCI: Comprehensive Complication Index
POMS: Postoperative Morbidity Survey
PAC: Preoperative Assessment Clinic
CGA: Comprehensive Geriatric Assessment
CAF: Comprehensive Assessment of Frailty
